# Quantitative susceptibility mapping shows lower brain iron content in children with attention‐deficit hyperactivity disorder

**DOI:** 10.1002/hbm.25798

**Published:** 2022-02-02

**Authors:** Shilong Tang, Guanping Zhang, Qiying Ran, Lisha Nie, Xianfan Liu, Zhengxia Pan, Ling He

**Affiliations:** ^1^ Department of Radiology Children's Hospital of Chongqing Medical University, National Clinical Research Center for Child Health and Disorders, Ministry of Education Key Laboratory of Child Development and Disorders, Chongqing Key Laboratory of Pediatrics Chongqing Chongqing China; ^2^ Department of Cardiovascular and Thoracic Surgery Children's Hospital of Chongqing Medical University Chongqing Chongqing China; ^3^ GE Healthcare, MR Research China Beijing China

**Keywords:** attention‐deficit hyperactivity disorder, brain, iron, magnetic resonance imaging, quantitative susceptibility mapping

## Abstract

To investigate the feasibility of quantitative susceptibility mapping in children with attention‐deficit hyperactivity disorder (ADHD), 53 children with ADHD aged 5–16 years were prospectively selected as the study group and 49 healthy children matched with age and gender were selected as the control group. All children underwent magnetic resonance imaging conventional sequence, 3D‐T1, and enhanced T2*‐weighted magnetic resonance angiography (ESWAN) sequence scanning. The iron content of brain regions was obtained through software postprocessing, and the iron content of brain regions of children with ADHD and healthy children was compared and analyzed to find out the characteristics of the iron content of brain regions of children with ADHD. The iron content in frontal lobe, globus pallidus, caudate nucleus, substantia nigra, putamen, and hippocampus of children with ADHD was lower than that of healthy children (*p* < .05). There was no significant difference in the content of iron in the left and right brain regions of children with ADHD (*p* > .05). The volume of frontal lobe and hippocampus of children with ADHD was lower than that of healthy children (*p* < .05). Iron content in brain areas such as globus pallidus, caudate nucleus, hippocampus, and putamen could distinguish children with ADHD (Area under curve [AUC] > 0.5, *p* < .05). Quantitative susceptibility mapping showed decreased iron content in some brain regions of children with ADHD.

## Graphical abstract

This study found that the decreased iron content in some brain regions of children with ADHD.

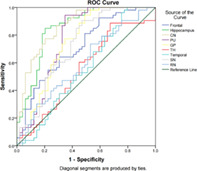

This study found that the globus pallidus, caudate nucleus, hippocampus, and putamen could distinguish children with ADHD (AUC > 0.5, *p* < .05).

**Abbreviations**
ADHD: attention‐deficit hyperactivity disorder
DSM‐V: Diagnostic and Statistical Manual of Mental Disorders, Fifth Edition
ESWAN: enhanced T2*‐weighted magnetic resonance angiography
MRI: magnetic resonance imaging
QSM: quantitative Susceptibility Mapping
ROC: receiver operating characteristic curve

## 1 BACKGROUND

Attention deficit hyperactivity disorder (ADHD) is one of the most common mental and behavioral disorders in childhood, characterized by inattention and hyperactivity. It is easy to be complicated with various behavioral problems such as learning difficulties, emotional disorders, and tic disorders, which seriously affect the academic and emotional development of children and adolescents, and have a great impact on family relations, partnership, and social function establishment (Aricò, Arigliani, Giannotti, & Romani, 2020; Becker, 2020; Craig, Bondi, O'Donnell, Pepler, & Weiss, 2020; Drechsler et al., 2020); Hoogman, Stolte, Baas, & Kroesbergen, 2020; Montagna et al., 2020; Slobodin & Masalha, 2020; Turan, Tunctürk, Çıray, Halaç, & Ermiş, 2021. ADHD is a multifactorial disease, which is related to nerve development, accumulation or lack of trace elements, and some factors have not been identified. For example, in recent years, researchers have found that the content of trace element iron in children with ADHD is lower than that in healthy children, but we do not know whether the decrease of trace element iron content leads to the decrease of iron content in brain area of children with ADHD (Doom et al., 2018; Lopez et al., 2019; Robberecht, Verlaet, Breynaert, De Bruyne, & Hermans, 2020; Wang, Huang, Zhang, Qu, & Mu, 2017)? Iron deficiency in the brain region can lead to hyperactivity symptoms such as mental laxity, irritability, and memory loss in the affected children (Arisaka, Ichikawa, Imataka, Koyama, & Sairenchi, 2020; Thirupathi & Chang, 2019; Vallée, 2017; Yu & Chang, 2019). How to accurately detect iron deficiency in the brain region of children with ADHD at an early stage by new technology so that they can be treated in time is one of the research hotspots for medical professionals.

Quantitative susceptibility mapping (QSM) is a new technology developed on the basis of susceptibility weighted Imaging in recent years to quantify the susceptibility distribution in biological tissues, and it uses magnetic resonance imaging phase information to detect magnetic sensitive substances and quantitatively calculate the magnetic susceptibility value of the substances. QSM technology can detect tiny changes in iron content in the brain, and has good specificity for spatial image comparison, with high sensitivity and reliability (Au et al., 2020; Chen et al., 2021; Tang et al., 2021; Uchida et al., 2019; Vinayagamani et al., 2020).

In this study, iron content values in each brain region of children were obtained by QSM technology, and iron content values in each brain region of children with ADHD and healthy children were compared and analyzed; the differences of iron content in each brain area of children with ADHD and healthy children were found, so as to accurately identify the characteristics of iron content in brain area of children with ADHD as soon as possible, so that it can be timely diagnosed and reasonably treated, improve the severity of children with ADHD's disease, and reduce the burden of family and society.

## 2 MATERIALS AND METHODS

This study was approved by the Ethics Committee of Children's Hospital Affiliated to Chongqing Medical University (*NO. 2019‐221*), and the family members of the study children signed an informed consent form before the examination.

### 2.1 Patient information

Study group: prospectively selected 68 children with ADHD from January 2020 to May 2021, and 53 children with ADHD were included in the study. Control group: prospectively selected 61 healthy children aged 5–16 years from February 2020 to June 2021, and 49 children were included in the study. Children who were not included in the study had abnormal brain lesions or did not cooperate with the sedation of the children, which caused the images to have motion artifacts.

Inclusion criteria for children in the control group: Children's body mass index is between 15 and 18 kg/m^2^; right‐handed, no neurological disease, no other organ‐related diseases, no other diseases that may affect brain function and structure, normal range of iron trace element detection in serum (6.5–9.85 μmol/L), there were no abnormal children in the routine head magnetic resonance examination.

Inclusion criteria for children in the study group: the children meet the diagnostic criteria of the American Diagnostic and Statistical Manual of Mental Disorders (DSM‐V) for ADHD; the first diagnosis of children with ADHD in the study group was performed at the outpatient clinic of a physician with an associate senior title or above in the Department of Child Developmental Behavior and Child Health and the Department of Psychology; and the children with ADHD included in the study met the diagnostic criteria for ADHD in the fifth edition of the Manual of Diagnosis and Statistics of Mental Disorders, the remaining inclusion criteria were the same as for the control group.

All the children who did not cooperate with the examination were sent to the sedation center of our hospital for examination after sedation. The sedation method was: taking dexamex 3 μg/kg nasal drop + chloral hydrate 40 mg/kg orally, and 20 min later, if the sedation depth was not enough, taking dexamex 1 μg/kg nasal drops again, and taking chloral hydrate 20 mg/kg orally if necessary.

### 2.2 Equipment and method

GE's 3.0 T magnetic resonance (GE Medical Systems, Milwaukee, WI) and 8‐channel head and neck joint coil were used. The uncooperative children were sedated and slept in the sedation center, and then underwent routine cranial MR sequence, 3D‐T1 and ESWAN sequence scanning, and routine sequence was T1 FLAIR, T2 FLAIR, T2 WI. 3D‐T1: TR, 450 ms; FOV, 25 cm; TE, 3.1 ms; NEX, 1 time; slice thickness, 1 mm; 152 slices; scanning time, 3 min 43 s. ESWAN: FOV, 24 cm; slice thickness, 3 mm; TR, 81.8; flip angle, 20; TE, 4 ms; Locs per slab, 50; slab, 1; scanning time, 7 min and 47 s.

#### 2.2.1 Data analysis

**FIGURE 1:**
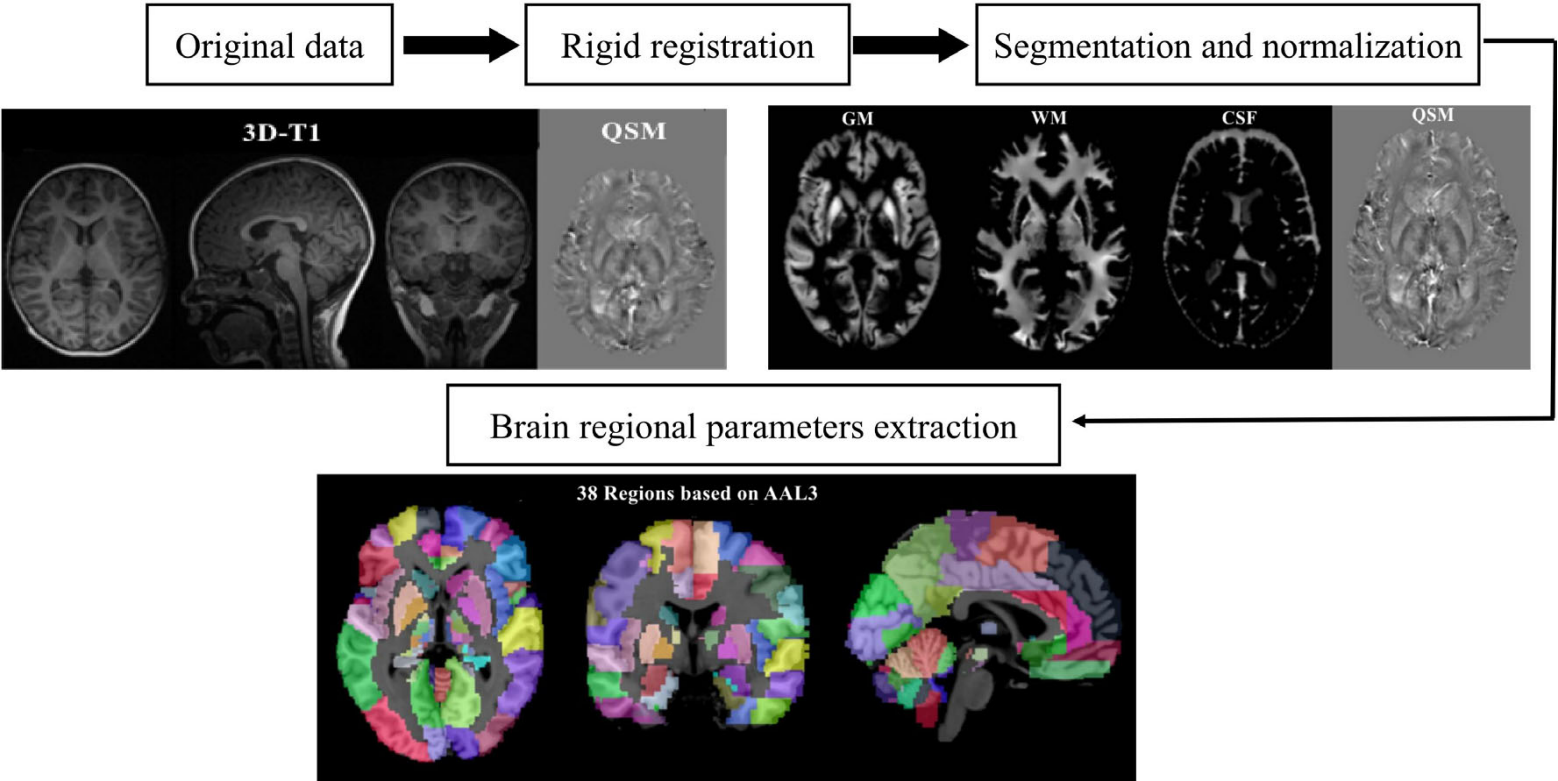
Schematic diagram of the image processing and parameter extraction. CSF, cerebrospinal fluid ; GM, gray matter; QSM, quantitative susceptibility mapping; WM, white matter

ESWAN raw data is used on MATLAB 2018a (MathWorks, Natick, MA) platform to obtain QSM quantitative map using STI Suite 3.0 software (https://people.eecs.berkeley.edu/chunlei.liu/software.html). In order to calculate the volume of different brain regions, QSM values, we adopted the voxel‐based morphometry (VBM) method. On the MATLAB 2018a platform, we use SPM12 software (http://www.fil.ion.ucl.ac.uk/spm/) to register the 3D‐T1 sequence structure map with the QSM quantitative map, and use the CAT12 toolkit (http://www.neuro.uni-jena.de/cat/) in the SPM12 software to segment the registered QSM structural quantitative map and, finally, extract each brain parameter values and iron content values (Figure 1).

### 2.3 Statistical analysis

Using SPSS 25.0 statistical software, measurement data are expressed as $\bar{x}\pm s$. The chi‐square test was used to compare the gender of children in the study group and the control group. Two independent samples *t* test was used to compare the age, weight, Body Mass Index (BMI) value, trace element iron detection value of the study group and the control group. Two independent samples t test was used to compare the brain volume of children between the study group and the control group (*p* < .05 means the difference is statistically significant). An analysis of covariance was performed using age as a covariate to compare the iron content in the brains of children in the study and control groups. The receiver operating characteristic curve (ROC) analysis is used to evaluate the diagnostic value of brain iron content in the diagnosis of children with ADHD. When the AUC is greater than 0.5 and statistically significant, it is considered to be of diagnostic value. The closer the value is to 1, the higher the diagnostic value.

## 3 RESULTS

### 3.1 Comparison results of patient information

There was no significant difference between the study group and the control group in age, gender, weight, BMI value, and trace iron detection value (*p* > .05) (Table 1).

**TABLE 1:** Patient information

Information group	Male‐to‐female ratio	Age	Weight	BMI	Trace iron
Control group	25:24	8.56 ± 2.54	28.15 ± 5.93	17.19 ± 1.87	7.38 ± 1.13
Study group	27:26	8.44 ± 2.13	29.23 ± 6.12	17.38 ± 2.06	7.24 ± 0.96
*X* ^2^/*T* value	0.000	0.255	−0.904	−0.486	0.676
*p* value	.994	.800	.368	.628	.501

### 3.2 Comparison results of brain iron content values

The iron content (total iron content in left and right brain regions)of the frontal lobe, globus pallidus, caudate nucleus, substantia nigra, putamen, hippocampus, and other brain regions of children with ADHD was lower than that of healthy children (*p* < .05); the difference in iron content between the left and right brain regions of children with ADHD was not statistically significant (*p* > .05); the brain total iron content of children with ADHD is lower than that of healthy children (*p* < .05) (Tables 2 and 3).

**TABLE 2:** Comparison of the iron content measurement data between the study and control groups [$\bar{x}$ ± s, ppb (×10^−9^)]

Brain regions group	Frontal	Hippocampus	CN	PU	GP	TH	Temporal	SN	RN
Control group	144.26 ± 23.33	169.62 ± 26.76	855.44 ± 133.89	76.44 ± 17.76	1,168.79 ± 236.71	369.58 ± 70.19	192.46 ± 49.13	549.41 ± 97.85	235.52 ± 46.07
Study group	125.75 ± 22.00	135.14 ± 19.04	651.25 ± 121.27	61.02 ± 10.57	996.19 ± 114.25	364.42 ± 81.17	184.85 ± 31.30	475.23 ± 69.94	221.10 ± 42.01
*F* ^#^ value	24.928	84.683	87.182	39.943	27.913	0.056	1.013	28.357	3.504
*p* value	<.001	<.001	<.001	<.001	<.001	.813	.317	<.001	.064

*Note*: An analysis of covariance was performed using age as a covariate to compare the iron content in the brains of children in the study and control groups (a corrected αlevel was taken to control for a total FDR less than 0.05, the number of comparisons was 9, and α' was set at .05/9 = .0056, that is, a *p* value less than .0056 was considered a statistically significant difference).

Abbreviations: CN, caudate nucleus; GP, globus pallidus; PU, putamen; RN, red nucleus; SN, substantia nigra; TH, thalamus.

**TABLE 3:** Iron content of brain regions in children [$\bar{x}$ ± s, ppb (×10^−9^)]

Group brain regions	Left	Right	*T* value	*p* value
Frontal	60.18 ± 13.24	61.93 ± 11.67	1.823	.984
Temporal	93.35 ± 16.41	94.58 ± 14.27	5.257	.092
Hippocampus	55.27 ± 18.32	56.89 ± 14.64	2.874	.143
TH	179.23 ± 23.43	178.26 ± 32.54	3.547	.752
GP	393.56 ± 78.89	392.47 ± 89.56	1.168	.247
SN	175.13 ± 38.03	176.98 ± 45.21	4.872	.993
PU	57.65 ± 13.26	56.18 ± 12.22	2.146	.109
RN	114.78 ± 26.67	113.89 ± 33.78	3.428	.136
CN	321.89 ± 79.27	322.96 ± 87.31	1.224	.143

Abbreviations: CN, caudate nucleus; GP, globus pallidus; PU, putamen; RN, red nucleus; SN, substantia nigra; TH, thalamus.

### 3.3 Brain volume comparison results

The brain areas of the frontal lobe and hippocampus of children with ADHD are lower than healthy children (*p* < .05); the total brain volume of children with ADHD is lower than that of healthy children (*p* < .05) (Table 4).

**TABLE 4:** Volume values of brain regions in children [$\bar{x\;}$± s, volume (cm^3^)]

Group brain regions	Control group	Study group	*T* value	*p* value
Frontal	189.77 ± 27.65	182.56 ± 23.53	12.871	.008
Temporal	138.32 ± 15.25	140.47 ± 18.34	10.132	.213
Hippocampus	12.37 ± 1.28	10.24 ± 1.56	7.401	.002
TH	14.97 ± 2.56	14.59 ± 2.39	8.601	.804
GP	3.62 ± 0.97	3.87 ± 1.13	1.354	.193
SN	0.94 ± 0.16	0.99 ± 0.26	4.457	.643
PU	16.97 ± 2.89	16.18 ± 3.45	3.871	.109
RN	0.72 ± 0.22	0.68 ± 0.13	7.422	.127
CN	9.26 ± 1.82	8.96 ± 1.25	3.214	.566
Whole brain	1452.89 ± 179.43	1437.96 ± 187.31	9.872	.995

Abbreviations: CN, caudate nucleus; GP, globus pallidus; PU, putamen; RN, red nucleus; SN, substantia nigra; TH, thalamus.

### 3.4 ROC analysis results

**FIGURE 2:**
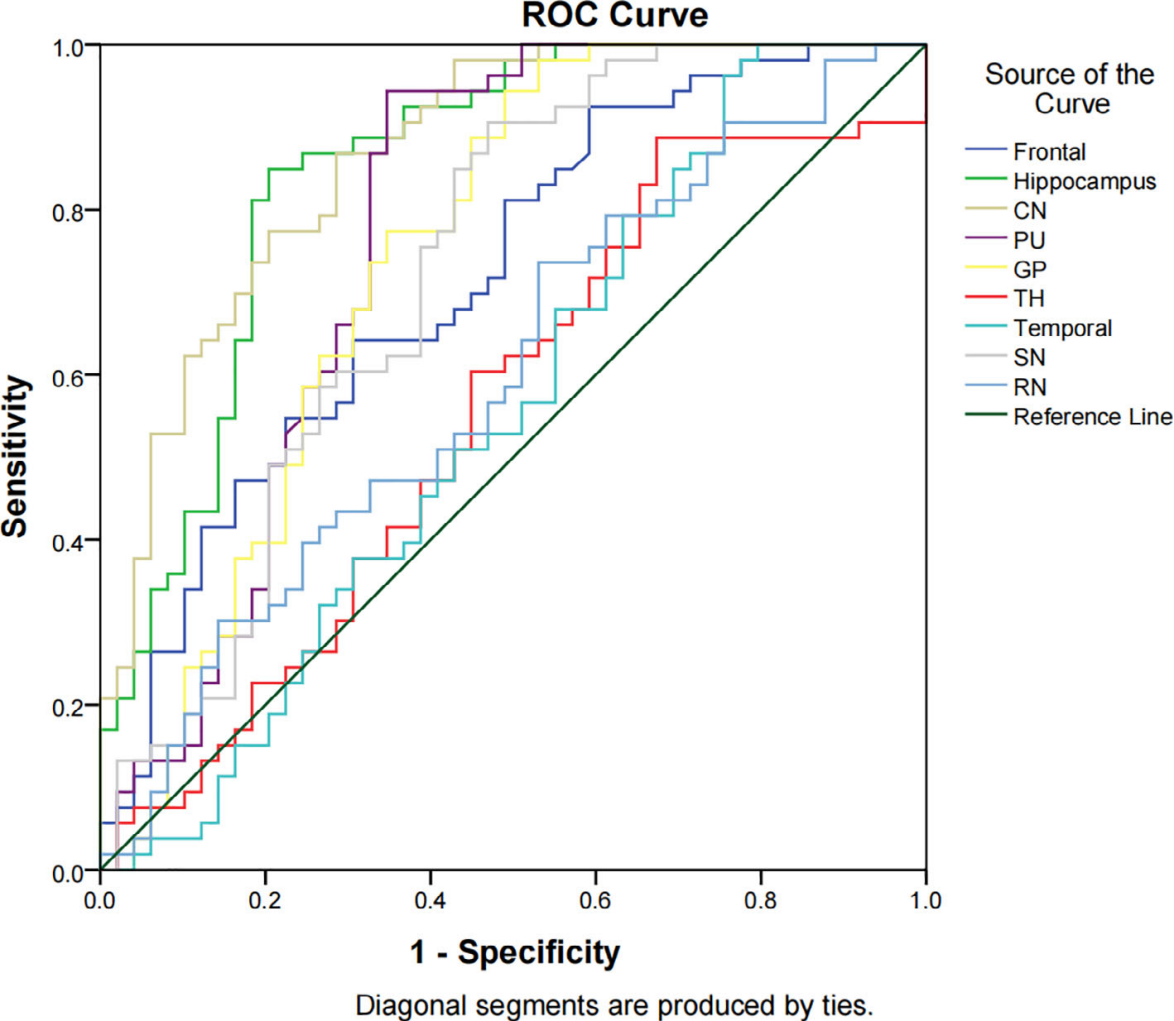
ROC curve analysis results of iron content in brain regions. CN, caudate nucleus; GP, globus pallidus; PU, putamen; RN, red nucleus; ROC, receiver operating characteristic curve; SN, substantia nigra; TH, thalamus

The iron content of the brain regions such as globus pallidus, caudate nucleus, hippocampus, and putamen can distinguish children with ADHD (AUC > 0. 5, *p* < .05), and the hippocampus AUC (0.881) value is the highest (Figure 2; Table 5).

**TABLE 5:** ROC curve analysis results of iron content in brain regions (*n* = 102)

Brain region	AUC	Std. error	*p* value	95% CI	
				Lower bound	Upper bound
Frontal	0.716	0.051	.503	0.617	0.815
Hippocampus	0.881	0.039	.000	0.775	0.927
CN	0.868	0.035	.000	0.799	0.936
PU	0.771	0.050	.000	0.673	0.870
GP	0.744	0.051	.000	0.644	0.844
TH	0.554	0.058	.347	0.441	0.668
Temporal	0.556	0.058	.326	0.442	0.671
SN	0.725	0.052	.102	0.624	0.827
RN	0.598	0.056	.090	0.487	0.708

Abbreviations: CI, confidence interval; CN, caudate nucleus; GP, globus pallidus; PU, putamen; ROC, receiver operating characteristic curve; RN, red nucleus; SN, substantia nigra; TH, thalamus.

## 4 DISCUSSION

Brain basal ganglia is an important neurological function area, which is closely related to sensory, motor, visual, and behavioral functions, and the basal ganglia is located in the deep part of the brain, where nerve cells are concentrated, mainly composing the caudate nucleus and putamen, globus pallidus, substantia nigra, etc. These neural structure and brain cortex and cerebellum common control and adjust the function of human body movement. Basal ganglia lesions mainly produce motor abnormalities (increased or decreased movement) and muscle tone changes (increased or decreased) (Bostan, Dum, & Strick, 2018; Pierce & Péron, 2020; Riva, Taddei, & Bulgheroni, 2018; Yanagisawa, 2018). The results (Table 2) showed that the iron content in the basal ganglia (globus pallidus, caudate nucleus, substantia nigra, and putamen) of children with ADHD was lower than that of healthy children (*p* < .05). The decrease of iron content may affect the development of nerve cells in the basal ganglia, resulting in abnormal neural development in the basal ganglia, and ultimately lead to ADHD and other abnormal behaviors in children.

The frontal lobe is the most developed brain lobe in the human body, including anterior, middle, and posterior regions, which is an important neural tissue region with extensive neural connections and complex structural diagrams. The functions of the frontal lobe include movement, memory, judgment, analysis and thinking, etc. Therefore, abnormalities of the frontal lobe can lead to abnormalities in movement, memory, and other aspects (Alsultan, Alaboudi, Almousa, Alajaji, & Bashir, 2020; Oades, 1998; Rolls et al., 2020; Ronan, Alexander‐Bloch, & Fletcher, 2020; Schmidt & Poole, 2020). The results show that the iron content in the frontal lobe of children with ADHD is lower than that of healthy children, and the decrease of iron content may also lead to neurodevelopmental abnormalities in the frontal lobe, and ultimately lead to behavioral abnormalities in children with ADHD.

The hippocampus is named for its similar shape to a seahorse, with a length of about 5 cm, mainly including the hippocampus finger, hippocampus head, dentate gyrus, and so on. It is closely related to learning, memory, emotion, movement, and other functions. Iron deficiency can affect neurometabolism in local brain regions, especially hippocampus (Cordier et al., 2021; Famitafreshi & Karimian, 2020; Kodali, Attaluri, et al., 2021; Kodali, Mishra, et al., 2021; Nelissen et al., 2017). Callahan, Thibert, Wobken, and Georgieff (2013) found that early iron deficiency can change the expression of key genes affecting synaptic plasticity in hippocampus, leading to electrophysiological abnormalities of cells and behavioral abnormalities of animals. The results show that the iron content in hippocampus of children with ADHD is lower than that of healthy children, the decrease of iron content may lead to abnormal hippocampal development, and ultimately lead to the abnormal mental activity of children with ADHD.

The results of the study found that children with ADHD only have abnormal brain areas such as the frontal lobe and hippocampus (lower than healthy children), but the areas with abnormal iron content include the frontal lobe, globus pallidus, caudate nucleus, substantia nigra, putamen, hippocampus, and other brain areas. The number of brain areas with abnormal iron content is more than the number of brain areas with abnormal volume, which means that abnormal brain areas in children with ADHD may not cause abnormal brain areas. The frontal lobe, hippocampus, and other brain regions of children with ADHD are lower than healthy children, which may be caused by the backward development of the frontal lobe, hippocampus, and other brain regions of children with ADHD than healthy children.

The results of the study found reduced iron content in frontal, temporal, and basal ganglia brain regions in children with ADHD, along with abnormalities in the volume of frontal and hippocampal brain regions (lower than healthy children), which is consistent with the findings of Chen et al. (lower brain volume in children with HDAD than healthy children) (Chen, Su, et al., 2021; Chen, Soldan, et al., 2021; Shi et al., 2021), suggesting that the reduced volume of frontal and hippocampal brain regions may be due to decreased iron content in brain regions (reduced iron content in brain regions leads to abnormal development of nerve cells in brain regions, and abnormal development of nerve cells leads to delayed development of brain regions, which eventually leads to decreased volume of brain regions). The normal volume of the basal ganglia brain regions and the decrease in the volume of the frontal and temporal lobes and other brain regions may be due to the fact that the frontal and temporal lobes and other brain regions are larger and developing faster in children, and the decrease in iron content leads to a delay in the development of the brain regions, which eventually leads to a significant decrease in the volume of the brain regions. The basal ganglia brain regions are normal in volume, probably because of their small size and early development (the child's brain develops from the bottom up, from the inside out, and from the back to the front). These brain regions (e.g., the nucleus accumbens, pallidum, etc.) do not change much in volume as children get older. Although decreased iron levels in these brain regions can lead to delayed development of brain regions, the differences are not significant compared to healthy children because of their small size. This ultimately resulted in a statistically insignificant difference in the volume of basal ganglia brain regions between children with ADHD and healthy children.

The results of the study found that the brain volume and iron content of the left and right sides of the children with ADHD were not statistically significant, suggesting that the abnormalities of the brain areas of the children with ADHD are symmetrical changes. The results of the study showed that the iron content of certain brain regions of children with ADHD was reduced, but the trace element iron test results were within the normal range, suggesting that changes in the iron content of the brain regions of ADHD may be more sensitive.

The results of the study in Figure 2 and Table 5 show that the iron content of brain regions such as globus pallidus, caudate nucleus, hippocampus and putamen can distinguish children with ADHD (AUC > 0.5, *p* < .05). Therefore, these brain regions can be used as the brains of children with ADHD key brain areas for imaging diagnosis.

### 4.1 Limitations of this study

This study is a single‐center study, and the results may be regional to some extent; fewer children with ADHD were included in the study, which may have some limitations, most of the children with ADHD included in this study were first‐time visitors to our psychology department and had not taken medication for psychiatric disorders, and only five children had been taking psychotropic medication for 1–3 months. The above deficiencies need to be further improved in the future research.

In conclusion, QSM technology can show reduced iron content in some brain regions of children with ADHD, which can distinguish children with ADHD and contribute to the diagnosis of children with ADHD.

## TRIAL REGISTRATION

This study protocol was registered at the Chinese clinical trial registry (ChiCTR2100046616).

## Data Availability

https://figshare.com/s/1c22e32887bff32467eb
